# Supplementary material for: Structural Insights into the Dynamic Assembly of a YFV sNS1 Tetramer
Source: Viruses. 2024 Jul 29;16(8):1212. doi: 10.3390/v16081212 (PMC11359903; doi:10.3390/v16081212)
Supplement: Supplementary file 1 [file viruses-16-01212-s001.zip › Supplmentary materials for Structural Insights into the Dynamic Assembly of YFV sNS1 Tetramer_PQ240622.pdf]

viruses MDPI

Supplementary Materials

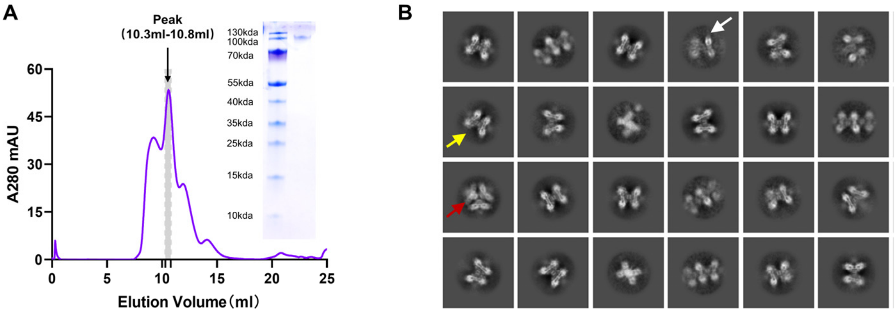

C

	Yfv_NS1 focused dimer	YFV_NS1 tetramer	YFV_NS1 hexamer
Percentage	42%	53%	5%

Figure S1. Purification of YFV sNS1.
(**A**) Size-exclusion chromatography and SDS-PAGE analysis of YFV sNS1. The peak of YFV sNS1 is indicated by the black arrow. (**B**) 2D class averages analysis of purified YFV sNS1. White, yellow and red arrows indicate the representative averages of flexible tetramer, tetramer and hexamer. (C) The percentage of the particles of flexible tetramer, tetramer and hexamer in 2D average classification.

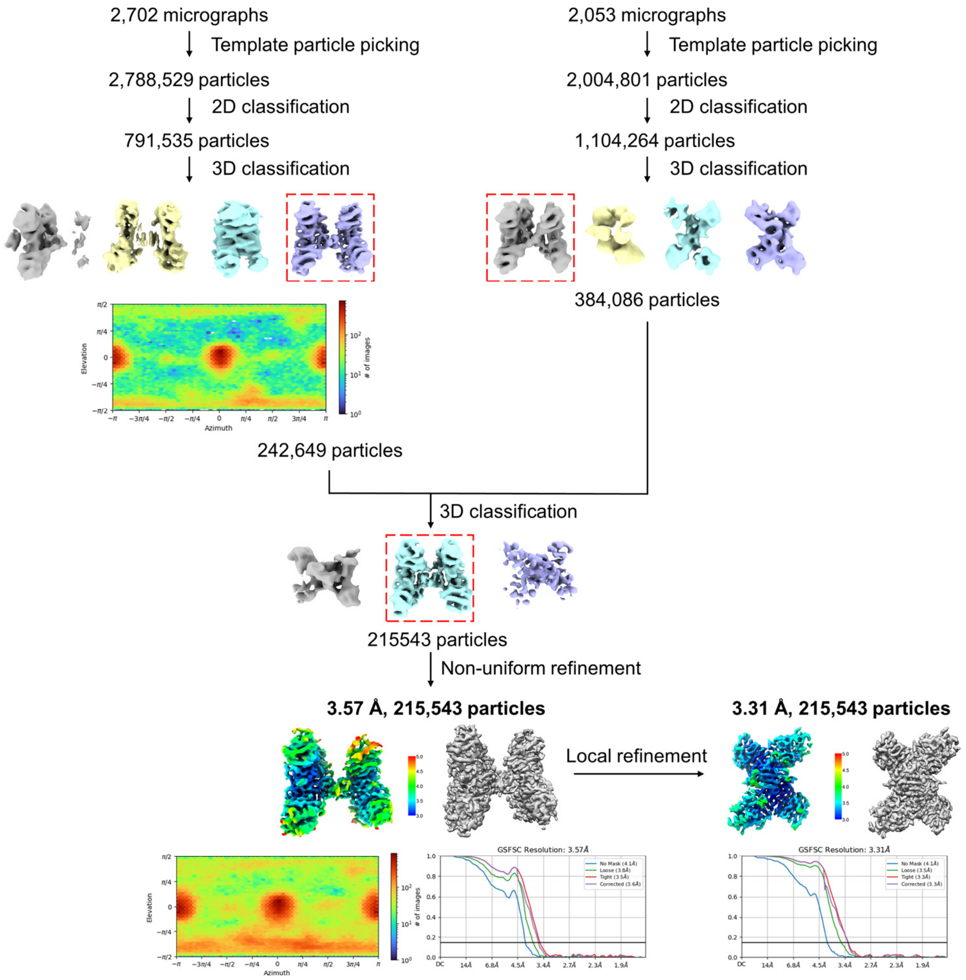

Figure S2. Data processing workflow for the reconstruction of YFV sNS1 dimer and tetramer.

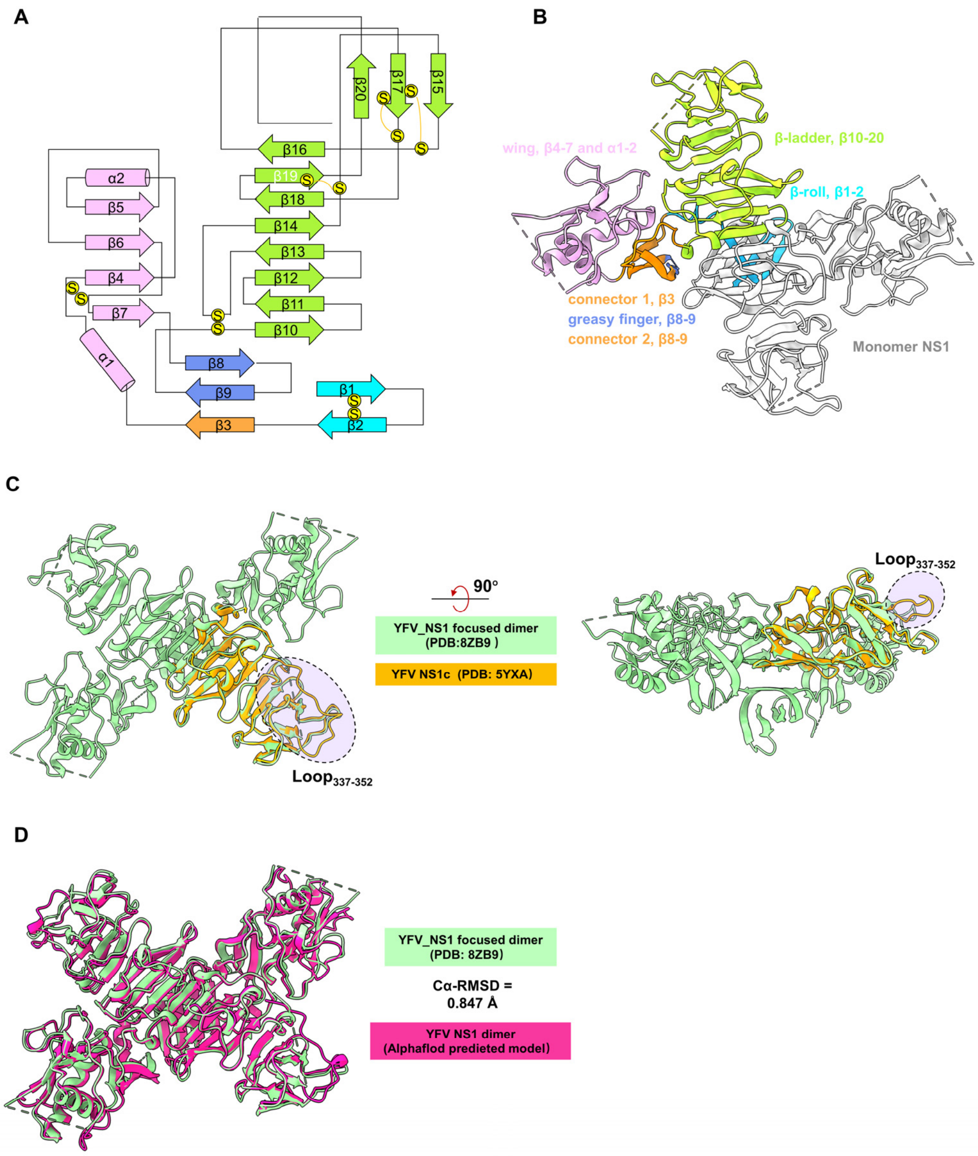

Figure S3. Structure scheme of YFV sNS1.
(**A-B**) Topology diagram of YFV sNS1 protomer and cartoon model of YFV sNS1 dimer. Both the topology diagram and one monomer cartoon model are colored by domains. β-roll: Deep sky blue; wing: Plum; β-ladder: Green yellow; connector domains: Dark orange; greasy finger: Royal blue; (**C**) Comparison of YFV NS1 dimer and C-terminal of YFV NS1 models. Cryo-EM structure of YFV NS1 dimer is colored in (green), and the crystal structure of YFV NS1c (PDB: 5YXA) is colored in (orange). Both peripheral view (left) and side view (right) are presented. Loop_337-352 are highlighted by circles. (**D**) The structural comparison between the YFV_NS1 focused dimer (light green) and 3D model of YFV NS1 dimer (hot pink).

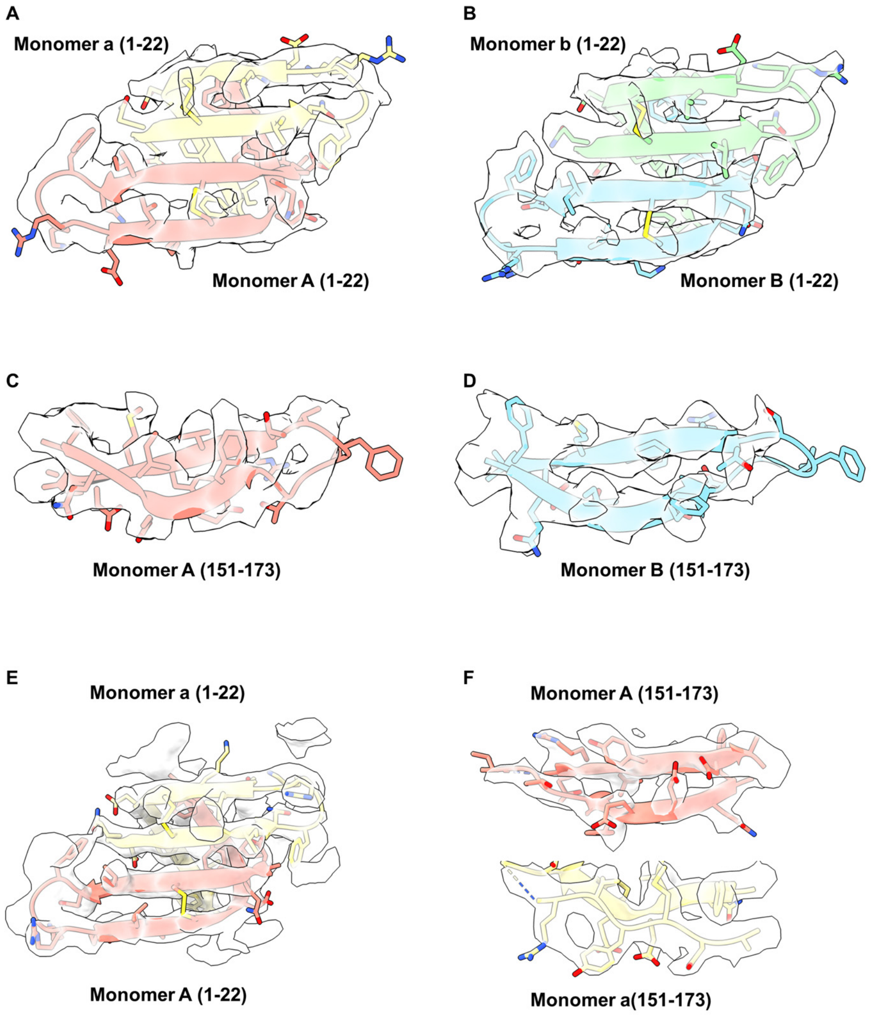

Figure S4. The local density maps of YFV_NS1_tetramer.
(**A**) The local density map of the β-roll domains of dimer Aa from YFV_NS1_tetramer. (**B**) The local density map of the β-roll domains of dimer Bb from YFV_NS1_tetramer. (**C**) The local density map of the greasy finger of monomer A from YFV_NS1_tetramer. (**D**) The local density map of the greasy finger of monomer B from YFV_NS1_tetramer. (**E**) The local density map of the β-roll domains of dimer Aa from YFV_NS1 focused dimer. (**F**) The local density map of the greasy finger of dimer Aa from YFV_NS1 focused dimer.

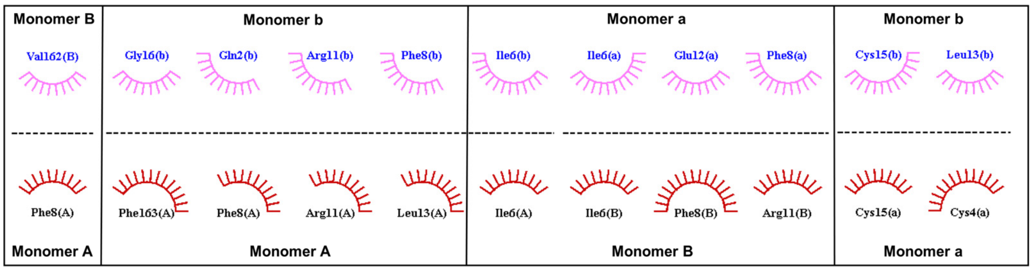

Figure S5. Interactions in the stacking zone of YFV NS1 tetramer.
The result is analyzed by LigPlot+.

A

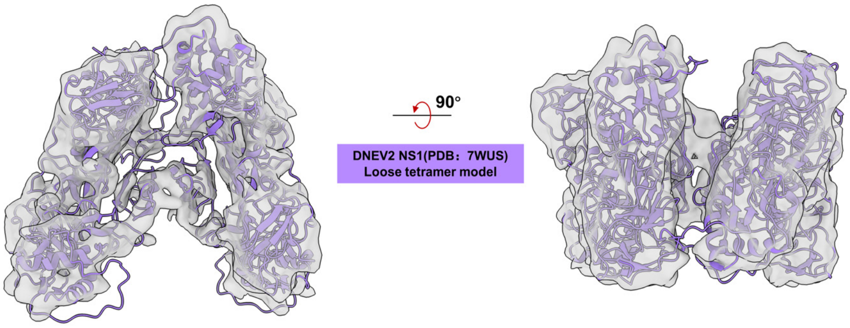

Figure S6. The model of DENV2 NS1 loose tetramer fitted by the structural of DENV2 NS1 dimer (PDB: 7WUS).

The atomic model is shown in cartoon representation and colored purple, and the density map is shown with 80% transparency.
